# Metabolomic analysis reveals the mechanism of aluminum cytotoxicity in HT-29 cells

**DOI:** 10.7717/peerj.7524

**Published:** 2019-08-27

**Authors:** Leilei Yu, Jiangping Wu, Qixiao Zhai, Fengwei Tian, Jianxin Zhao, Hao Zhang, Wei Chen

**Affiliations:** 1 School of Food Science and Technology, Jiangnan University, Wuxi, China; 2 International Joint Research Laboratory for Probiotics, Jiangnan University, Wuxi, China; 3 State Key Laboratory of Food Science and Technology, Jiangnan University, Wuxi, China; 4 (Yangzhou) Institute of Food Biotechnology, Jiangnan University, Yangzhou, China; 5 National Engineering Research Center for Functional Food, Jiangnan University, Wuxi, China; 6 Beijing Innovation Centre of Food Nutrition and Human Health, Beijing Technology & Business University, Wuxi, China

**Keywords:** HT-29 cell, Heavy metal, Metabolomic, Aluminum, Cytotoxicity

## Abstract

**Background:**

Aluminum (Al) is toxic to animals and humans. The most common sources of human exposure to Al are food and beverages. The intestinal epithelium is the first barrier against Al-induced toxicity. In this study, HT-29, a human colon cancer cell line, was selected as an in vitro model to evaluate the Al-induced alteration in metabolomic profiles and explore the possible mechanisms of Al toxicity.

**Methods:**

MTT assay was performed to determine the half-maximal inhibitory concentration of Al ions. Liquid chromatography-mass spectrometry (LC-MS) was used for metabolomic analysis, and its results were further confirmed using quantitative reverse transcription polymerase chain reaction (RT-qPCR) of nine selected genes.

**Results:**

Al inhibited the growth of the HT-29 cells, and its half-maximal dose for the inhibition of cell proliferation was found to be four mM. This dose was selected for further metabolomic analysis, which revealed that 81 metabolites, such glutathione (GSH), phosphatidylcholines, phosphatidylethanolamines, and creatine, and 17 metabolic pathways, such as the tricarboxylic acid cycle, pyruvate metabolism, and GSH metabolism, were significantly altered after Al exposure. The RT-qPCR results further confirmed these findings.

**Conclusion:**

The metabolomics and RT-qPCR results indicate that the mechanisms of Al-induced cytotoxicity in HT-29 cells include cellular apoptosis, oxidative stress, and alteration of lipid, energy, and amino acid metabolism.

## Introduction

Aluminum (Al), a metal that is toxic to animals and humans, is ubiquitous in industrialized societies, and human exposure to Al in daily life is inevitable. The major sources of human exposure to Al include food additives, food and beverage packaging, drinking water, and cooking utensils (Pineton de Chambrun et al., 2014; EFSA, 2008). Orally ingested Al is absorbed via the intestine. Reportedly, Al intake exceeds the health-based guidance value in the general population worldwide, especially in children, the most vulnerable and sensitive population (Vignal, Desreumaux & Body-Malapel, 2016). The human intestinal tract constitutes the largest interface between the human body and the external environment and is one of the primary barriers against the external environment (Kaminsky & Zhang, 2003; Salim & Söderholm, 2011). It has been reported that 38% of ingested Al accumulates in the intestinal mucosa and exerts negative effects on gut homeostasis (Cunat et al., 2000; Powell et al., 1994), such as alteration of gut permeability, dysbiosis of gut microbiota, and impairment of intestinal immune function (Vignal, Desreumaux & Body-Malapel, 2016; Yu et al., 2016). Ingestion of 50–475 mg/kg Al for approximately 2 months have been reported to induce histological alterations, such as mucosal degeneration, lymphocyte proliferation, and focal infiltration of monocytes in the lamina propria, in the small intestine of rodents (Al-Qayim & Saadoon, 2013; Perl et al., 2004; Roszak et al., 2017). Exposure to even low doses of Al (1.5 mg/kg) has been shown to aggravate intestinal damage in mouse colitis models (Pineton de Chambrun et al., 2014), such as worsening of colitis and upregulation of the mRNA expression of inflammatory cytokines IL1β, IL17A, and Nlrp3. Moreover, Al treatment has been shown to suppress the growth of murine intestinal bacteria (Lerner et al., 2006). Perl et al. (2004) hypothesized that Al can gain access to microorganisms through metal-chelating systems and then increase their pathogenicity and ability to induce an exuberant granulomatous response. Al was found to exert deleterious effects on the intestinal barrier integrity by inhibiting epithelial cell proliferation (both in vivo and in vitro), inducing bacterial translocation in mesenteric lymph nodes, and decreasing intercellular junction expression in the colon (Pineton de Chambrun et al., 2014). Al is also considered as an environmental trigger for a group of intestinal disorders represented by inflammatory bowel diseases (Lerner, 2007; Perl et al., 2004).

Although several studies have focused on the mechanisms underlying Al-induced intestinal injuries, the association between the altered molecular profile of cells and Al exposure has not yet been clarified. Metabolism is the foundation of all living systems. Metabolites, the end products of cellular processes, reflect the system-level biological stress response. Hence, alterations in metabolites are directly or indirectly related to changes in cellular processes or metabolism (Tripathi et al., 2008). Metabolomics, the newest “omics” discipline, provides a comprehensive view of metabolic fluxes and a unique insight into the environment of cells (Booth et al., 2011). For metal toxicity studies, metabolomics may be used to identify changes in metabolic pathways that would be missed by other omics techniques. For example, levels of proteins and mRNA in the cell do not necessarily translate to changes in the metabolic pathways and cannot reflect changes in the metabolic flux. Metabolomics promises to fill this gap and provide information not accessible through other techniques (Booth et al., 2011). Metabolomics covers the identification and quantification of endogenous low-molecular-weight metabolites (less than ~1,000 Da), which include small compounds such as nucleotides, lipids, and amino acids (Madji Hounoum et al., 2015). In general, potential molecular biomarkers discovered by the clarification of metabolic changes can serve as a basis for the comprehensive study of intestinal injury caused by metal exposure. For example, using metabolomics, Tripathi et al. (2008) showed that after 90 days of Al treatment, the levels of citric acid, creatinine, allantoin, succinic acid, alanine, glutamine, β-hydroxy-butyrate, and acetoacetate in rat serum and urine significantly reduced and those of acetate and acetone significantly increased. These overall perturbations observed in the metabolic profile demonstrated the impairment in the tricarboxylic acid (TCA) cycle and liver and kidney metabolism. Similarly, Lu et al. (2017) analyzed the effects of a traditional Chinese medicine Shengmai San (SMS) on Al-induced Alzheimer’s disease using metabolomics and showed that lipid peroxidation was the main mechanism of SMS intervention in Alzheimer’s disease, including inhibition of linoleic acid hydroperoxide generation. Among the several metabolomics methods available, liquid chromatography-mass spectrometry (LC-MS) was selected for use in our study because it has broad metabolite coverage and high sensitivity, as well as simple sample preparation.

The global reactions induced in intestine cells by Al exposure are still unclear. HT-29, a human colon cancer cell line, produces the secretory component of IgA, carcinoembryonic antigen, and mucins, which are suitable for heavy metal toxicity study due to the disturbance of metals on intestinal immune aspects (Hsu et al., 2017; Aicher et al., 1990; Forsberg et al., 2004). Many studies investigating the mechanisms of metal toxicity in intestinal cells by metabolomics or proteomics analyses have used HT-29 cells (Ezhilarasi et al., 2016; Lee et al., 2012; Brown, Yedjou & Tchounwou, 2008). In addition, our previous study showed that HT-29 cells are responsive to Al (Yu et al., 2016). Therefore, in this study, we investigated the Al exposure-induced global metabolic changes in the HT-29 cell line using LC-MS and gene expression analysis. The results improve our understanding of the mechanism of interaction between intestinal cells and Al toxicity and aid in further exploring strategies to alleviate Al toxicity.

## Materials and Methods

### Chemicals

Aluminum chloride was obtained from Sigma-Aldrich, St. Louis, MO, USA. TRIzol was bought from Ambion, Life Technologies (Grand Island, NY, USA). The iTaq™ universal SYBR^®^ Green one-step kit was purchased from Bio-Rad (Berkeley, CA, USA). All of the materials used for cell experimentation were bought from Shanghai Chemical Reagent Company (Shanghai, China). MTT and dimethyl sulfoxide (DMSO) used for detecting cell viability were purchased from Gibco, Life Technologies (Grand Island, NY, USA). Milli-Q AdvantageA10 used to provide ultrapure water was obtained from Millipore (Boston, MA, USA).

### Cell culture

HT-29, the human colon cancer cell line, acquired from the Shanghai cell bank of the Chinese Academy of Sciences, was cultured in RPMI-1640 medium supplemented with 10% fetal bovine serum and 1% penicillin–streptomycin at 37 °C in a 5% CO_2_ atmosphere. The cell culture was passaged every other day by trypsinizing the cells and diluting them at 1:3/1:2 in fresh medium to start a new cycle. The cells were then used in the following analysis when they reached the logarithmic growth phase.

### Metabolic activity detection

MTT assay for cell viability was performed to determine the half-maximal inhibitory concentration (IC50) of Al ion (Nzengue et al., 2008; Xu et al., 2010). Briefly, the cells in the logarithmic growth phase were added to a 96-well plate at a density of 103–104 cells per well. Once each well was coated with a monolayer, fresh medium supplemented with sterile phosphate-buffered saline (PBS; filtered through a 0.22-μm-pore size Millipore filter) containing Al ions at a final concentration of 0, 2, 4, 6, 8, or 10 mM was added to separate wells. After 24-h incubation, 20 μL of five mg/mL MTT was added to each well, and the cells were incubated for another 4 h. Subsequently, the medium in each well was replaced with 150-μL DMSO, and the cell viability was determined by measuring the absorbance of well contents at 490 nm.

### Cell collection and cell extraction

The Al ion concentration of four mM was selected for further analyses based on the results of our MTT assay and of a previous study (Pineton de Chambrun et al., 2014). HT-29 cells were seeded at a density of 106 cells per well in medium containing four mM Al ion and were incubated for 24 h. Cells incubated in medium devoid of Al ion were considered as control. After incubation, the cells were washed twice with PBS and frozen in liquid nitrogen for quenching metabolism. The procedure of extraction reported in a previous study was used with slight modifications (Van den Eede et al., 2015). Briefly, one mL of trichloromethane–water–methanol mixture at a ratio of 1:1:4 (v/v/v) was added to the wells for 5 min. The cells were then detached from the wells using sterile rubber scrapers and transferred to precooled bottles (−20 °C). Each well was rinsed with another one mL of extraction solution, and the residue was also transferred to the bottle. The samples were vortexed and subjected to ultrasonication in an ice-water bath at 300 W for 3 min (6-s/4-s on/off pulses). The samples were then transferred into 1.5-mL Eppendorf tubes and subjected to supersonic extraction for 3 min, followed by centrifugation at 15,000 rpm for 10 min (4 °C). The supernatant (200 μL) was used for metabolomic analysis by LC-MS.

### Metabolomic analysis

Agilent 1290 Infinity Ultra High-Performance Liquid Chromatography (UHPLC) system combined with Agilent 6538 UHD and Accurate-Mass Q-TOF/MS were used for data processing in the positive and negative ionization modes. The parameters of UHPLC and MS were further optimized based on a previous study (Van den Eede et al., 2015) to meet the needs of the current conditions. A C_18_ column (Waters ACQUITY UPLC@HSS T3; 100 × 2.1 mm, 2.1 μm) was used to separate the samples. Solvent A was 0.1% (v/v) formic acid in water, and solvent B was 0.1% (v/v) formic acid in acetonitrile. Each time, a three μL sample was injected into the column maintained at 40 °C. The liquid flow rate was 0.4 mL/min, with the following gradient conditions: 0 min, 5% B; 2 min, 5% B; 13 min, 95% B; 15 min, 95% B.

The capillary voltages used for the negative- and positive-ion modes were 3,500 V and 4,000 V, respectively. The reference masses 112.985587 m/z and 1033.988109 m/z were used in the negative mode and 121.0509 m/z and 922.0098 m/z were used in the positive mode. A quality control sample was injected every eight samples to ensure consistent quality.

### Data treatment

Agilent Mass Hunter Qualitative software was used for data conversion. Peak alignment and integration were conducted using XCMS (https://metlin.scripps.edu/) (Smith et al., 2006). A 3D matrix with retention time (RT), peak intensity, and m/z were obtained. The following XCMS parameters were used: profmethod = bin, method = matched Filter, step = 0.1, full width at half maximum (peak width) = 8, bandwidth = 10, snthresh = 5, ppm = 20, mzdiff = 0.01, and minfrac = 0.8. Mass range was 50–1100 m/z, mass tolerance was 20 ppm, RT range was 0.5–15.0 min, and RT width threshold was 0.2 min. The following formula was used to integrate the area of the peaks: unimodal area/area of all peaks in a single sample × 10,000. The SIMCA-P+ 14.0 software was used for principal component analysis (PCA) and partial least squares-discriminant analysis (PLS-DA). Two principle components for the positive mode and two for the negative mode were used in PCA. Five principal components for the positive mode and four for the negative mode were used in PLS-DA. Variables with variable importance in projection (VIP) values of >1 were considered as differential variables. Seven-round cross-validation with 200 response permutation tests was used to prevent model overfitting and determine the model quality.

### Metabolite screening

Metabolites were screened using univariate and multivariate analyses, the screening criteria (VIP > 1, *P* < 0.05, fold change > 1.5 or < 0.67), and the mass tolerance threshold (20 mDa). The HMDB and METLIN databases were used for metabolite identification, and the MBRole database was used for pathway enrichment.

### Verification of key enzymes by RT-qPCR

The relative expressions of nine related genes in the altered metabolomics pathways were further validated using quantitative reverse transcription polymerase chain reaction (RT-qPCR). The corresponding encoded proteins were found to be succinate dehydrogenase (sdhA), citrate synthase (CS), isocitrate dehydrogenase (IDH), lactic dehydrogenase (LDH), pyruvate dehydrogenase (PDH), pyruvate kinase (PK), glutamic oxaloacetic transaminase (GOT), glutathione peroxidase (GPx), and glutathione reductase (GR). The genes of the first three, middle three, and last three proteins are related to the TCA cycle, glycolysis, and glutathione (GSH) metabolism, respectively. Total RNA was extracted using the traditional TRIzol method. TAKARA RR047A kits were used to obtain cDNA by reverse transcription. Subsequently, 20-mL reaction mixture was prepared by combining cDNA, 2× Bio-Rad iTaq™ Universal SYBR^®^ Green Supermix, and specific primer pairs (Table 1) and subjected to RT-qPCR. Each reaction had three parallels, and the β-actin gene was used as the internal reference gene.

**Table 1:** Sequences of primers used in RT-qPCR.

Primer	Sequence (5′–3′)
PDH-F	TCAACTACCTGGTGCTTCG
PDH-R	CATCTCCAAATGCCCTAA
sdhA-F	ATTAACAGTCAAGGCGAAAG
sdhA-R	ACAACCAGGTCCAAGAGC
GPx-F	CCAGTCGGTGTATGCCTTCT
GPx-R	GATGTCAGGCTCGATGTCAA
GR-F	GCTGATTAAAGCTTTCCAAGTTGTG
GR-R	GTAAAGCTCGAGGAATAGGTCTTCAC
GOT-F	ACTCAAGGAGAAGCGGGTAG
GOT-R	ACAGGCGTGGAGGACAAC
CS-F	CGTTTCCGAGGCTTTAGT
CS-R	ACAAGGTAGCTTTGCGATT
IDH-F	CACTACCGCATGTACCAGAAAGG
IDH-R	TCTGGTCCAGGCAAAAATGG
LDH-F	TGTGCCTGTATGGAGTGG
LDH-R	TTATTCCGTAAAGACCCT
PK-F	GCACGCCAAGTACAACACC
PK-R	CACGCTCCCACATTCCATA
β-actin-F	GGGACCTGACTGACTACCTC
β-actin-R	TCATACTCCTGCTTGCTGAT

**Note:**

PDH, pyruvate dehydrogenase; sdhA, succinate dehydrogenase; GPx, glutathione peroxidase; GR, glutathione reductase; GOT, glutamic oxaloacetic transaminase; CS, citrate synthase; IDH, isocitrate dehydrogenase; LDH, lactic dehydrogenase; PK, pyruvate kinase.

The relative amounts of mRNA were calculated using the following equation:

$${\rm{Relative}}\,{\rm{expression}}\,{\rm{ = }}\,{{\rm{2}}^ \wedge }\, - \,\left[ {\left( {{\rm{C}}{{\rm{T}}_{{\rm{AT}}}}\, - \,{\rm{C}}{{\rm{T}}_{{\rm{AR}}}}} \right)\, - \,\left( {{\rm{C}}{{\rm{T}}_{{\rm{CT}}}}\, - \,{\rm{C}}{{\rm{T}}_{{\rm{CR}}}}} \right)} \right],$$

where AT is the target gene in the Al-treated group, AR is the reference gene in the Al-treated group, CT is the target gene in the control/untreated group, and CR is the reference gene in the control group.

## Results

### MTT assay for cell viability

**Figure 1:**
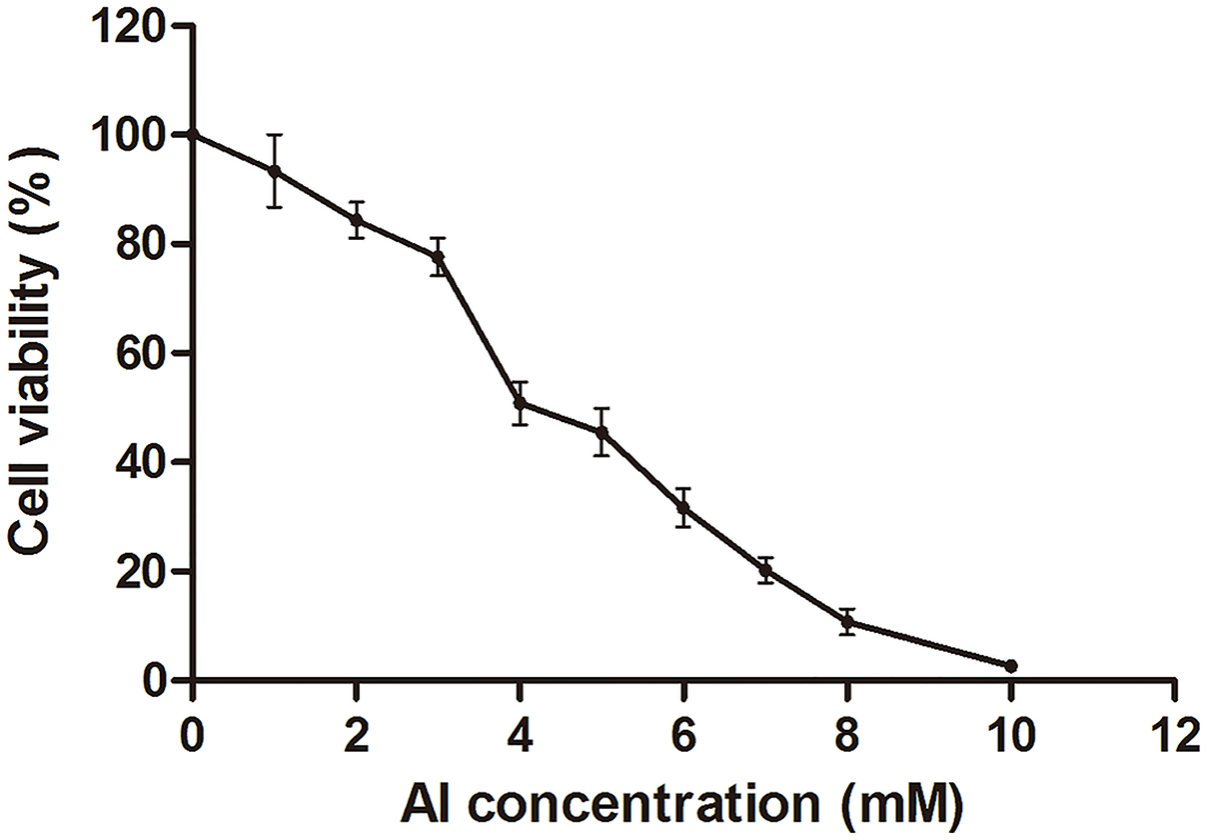
Cell viability of HT-29 cells after Al exposure. Viability is expressed as a cell activity percentage between the Al group and the control group. The experimental data are expressed as the mean value ± SD of six independent replicates.

The HT-29 cell viability decreased with the increase in Al concentration from 0 to 10 mM (Fig. 1; Dataset S1). Cell viability decreased to approximately 50% at four mM of Al ion concentration. This concentration was used to study Al cytotoxicity in the subsequent experiment, consistent with that in a previous study (Pineton de Chambrun et al., 2014).

### Metabolomic characterization of cells

The metabolites in the Al-treated group were compared with those in the control group using typical base peak intensity chromatograms. The results are presented in Fig. S1 for both the positive- and negative-ion modes. In total, 995 and 2,272 discrepant metabolites in the negative and positive modes, respectively, were selected by UPLC/MS for further analysis.

**Figure 2:**
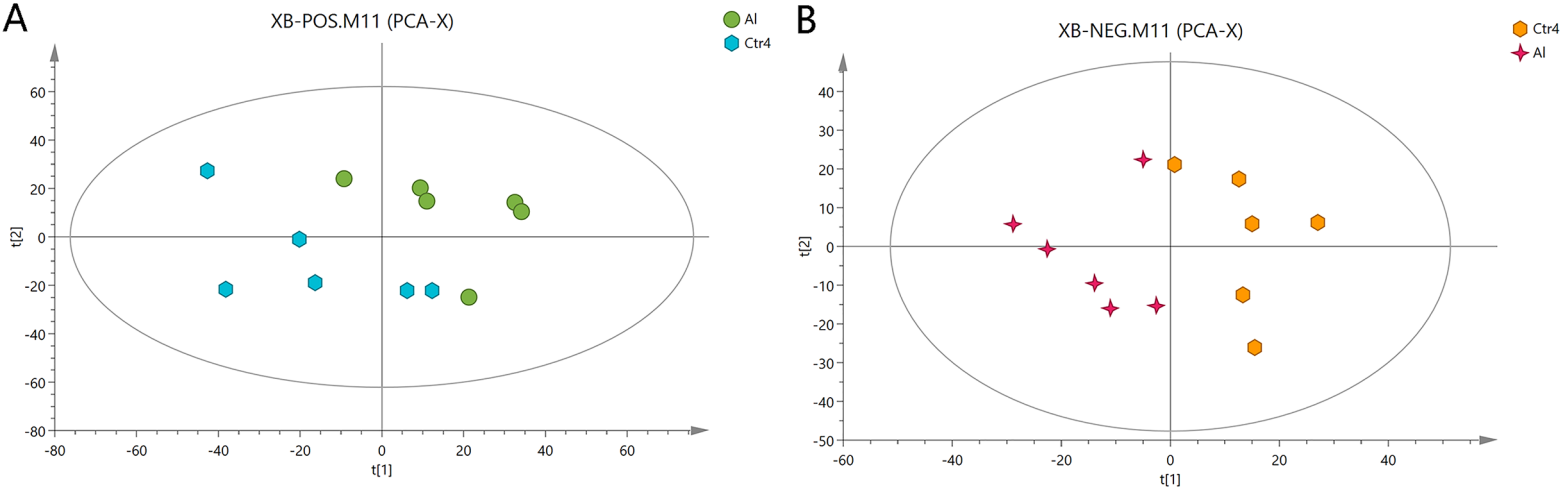
PCA score plots of metabolites in HT-29 cells in the positive-ion mode (A) and negative-ion mode (B).

First, unweighted analysis was used to evaluate the positive and negative data. The relationships of the metabolites between the Al-treated and control groups were identified by comparative analysis. The R2X, a representative parameter of the PCA model, was 0.472 and 0.548 for the positive and negative modes, respectively. The PCA score plots in Fig. 2 show that six replicates of the Al-treated group were separated from the samples in the control group. This result indicates that Al exposure caused some alteration in the metabolic profile of HT-29 cells.

### Extraction of special metabolites

**Figure 3:**
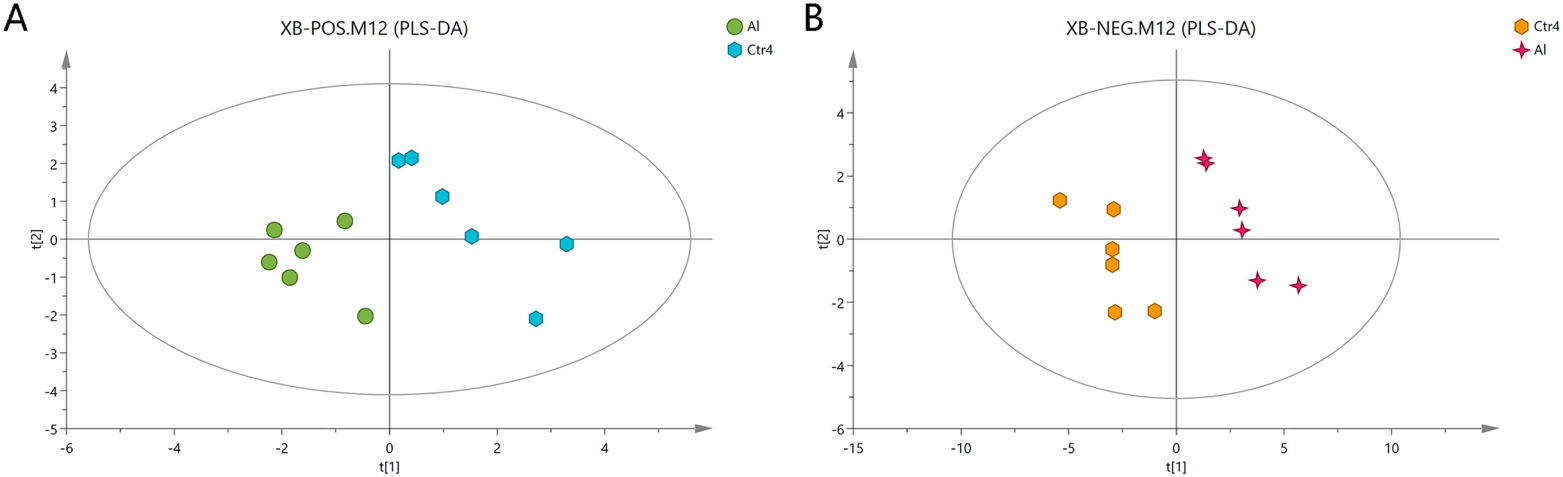
PLS-DA score plots of metabolites in HT-29 cells in the positive-ion mode (A) and negative-ion mode (B).

The Al-treated cells and untreated cells were further analyzed by PLS-DA. The more closely the three indexes (R^2^X, R^2^Y, and Q^2^) approached 1, the more credible and predictable are the metabolomics data. As shown in Fig. 3, in the above case, the R^2^X values were 0.725 and 0.745, the R^2^Y values were 1 and 0.997, and the Q^2^ values were 0.474 and 0.823 for the positive- and negative-ion modes, respectively. Partial least squares-discriminant analysis was cross-validated, and the results are shown in Fig. S2. All of the metabolites were judged by their VIP values, which reflected the proportion of their influence on the model.

The following screening criteria were rigorously applied to screen the metabolites as potential biomarkers: fold change > 1.5 or < 0.667, VIP > 1, and *P* < 0.05 (relative peak areas of the metabolite contents). The METLIN and HMDB databases were used to screen and characterize the candidates, which were then annotated as 39 and 42 metabolites in the positive- and negative-ion modes, respectively (Tables 2 and 3; Dataset S2). The metabolites GSH, glutamic acid, four phosphatidylcholines (PC (14:1), PC (14:0), PC (15:0), and PC (14:1)), phosphatidylethanolamine (PE (22:4)), pantothenate, creatine, and choline, were consistently downregulated after Al exposure.

**Table 2:** Altered metabolites of HT-29 cells after Al exposure in the positive-ion mode.

No.	Metabolites	M/Z	RT (min)	VIP value	*P* value	FC
1	MG (18:2(9Z,12Z)/0:0/0:0)	355.28	13.29	1.33	0.012	0.15
2	PC (14:1(9Z)/P-16:0)	688.52	12.14	1.30	0.002	0.29
3	Beta-D-3-Ribofuranosyluric acid	301.08	14.35	1.30	0.005	0.29
4	N-Acryloylglycine	130.05	0.85	1.29	0.013	0.30
5	Beta-D-Glucopyranosyl-11-hydroxyjasmonic acid	389.18	0.73	1.28	0.001	0.33
6	Vaccenyl carnitine	426.36	10.69	1.27	0.001	0.34
7	8-Hydroxypinoresinol 8-glucoside	537.20	0.67	1.27	0.002	0.42
8	Creatine	263.15	0.73	1.27	0.000	0.42
9	SM (d18:1/14:0)	675.54	13.16	1.25	0.000	0.43
10	PC (o-4:0/16:0)	692.56	14.95	1.25	0.007	0.43
11	L-Glutamic acid	148.06	0.72	1.24	0.000	0.48
12	LysoPC (18:0)	524.37	11.96	1.23	0.027	0.48
13	Caffeoylcycloartenol	589.43	13.79	1.23	0.000	0.49
14	Tetradecanoylcarnitine	372.31	9.67	1.22	0.000	0.51
	Myristic acid	267.17	11.01	1.22	0.015	0.51
15	3-Hydroxyphenyl-valeric acid	233.06	1.03	1.20	0.009	0.53
16	N-Hexadecanoylpyrrolidine	619.61	14.81	1.20	0.005	0.53
17	LysoPE (18:0/0:0)	482.32	11.91	1.20	0.001	0.53
18	Phenylpyruvic acid	165.05	1.17	1.19	0.003	0.54
19	LysoPC (18:2(9Z,12Z))	520.34	10.17	1.18	0.000	0.56
20	Glyceryl lactooleate	429.32	12.51	1.17	0.003	0.57
21	Methylgingerol	309.21	11.35	1.17	0.004	0.57
22	Palmitoleoyl ethanolamide	298.27	11.75	1.17	0.026	0.57
23	PC (15:0/18:1(11Z))	746.57	12.81	1.17	0.004	0.58
24	7-Hydroxydehydroglaucine	392.15	3.87	1.17	0.035	0.58
25	UDP-N-acetyl-alpha-D-galactosamine	630.07	0.80	1.16	0.001	0.59
26	Adrenoyl ethanolamide	376.32	12.78	1.16	0.041	0.60
27	Meta-O-Dealkylated flecainide	371.10	14.24	1.15	0.041	0.60
28	L-Agaritine	535.26	9.81	1.15	0.009	0.61
29	PE (22:4(7Z,10Z,13Z,16Z)/15:0)	754.54	14.95	1.14	0.033	0.62
30	Alpha-CEHC	279.16	11.98	1.14	0.003	0.63
31	Propionylcarnitine	218.14	1.49	1.13	0.003	0.63
32	Glycerol 1-hexadecanoate	331.28	13.71	1.13	0.028	0.63
33	Palmitoleoyl ethanolamide	320.26	10.49	1.13	0.002	0.63
34	Isolimonic acid	507.22	9.05	1.13	0.010	0.64
35	PC (22:2(13Z,16Z)/14:1(9Z))	784.58	13.31	1.12	0.047	0.64
36	6,10,14-Trimethyl-5,9,13-pentadecatrien-2-one	263.24	12.90	1.11	0.015	0.65
37	4-Hydroxymidazolam	364.06	0.76	1.11	0.019	0.65
38	Alpha-CEHC	301.14	11.98	1.11	0.036	0.65
39	MG (18:0e/0:0/0:0)	367.32	12.39	1.09	0.005	0.66

**Notes:**

M/Z, mass/charge number of peaks in the mass spectra; RT, retention time of metabolites in chromatography; FC, fold change of metabolites after Al exposure. Differences between the control and Al-treated cells were analyzed by one-way analysis of variance, followed by the Tukey’s post hoc test. *P* < 0.05 was considered as significant. The changes in metabolite abundance are expressed as the ratio of the average content in the treatment and control groups (*n* = 6). A value < 1 indicates downregulation.

**Table 3:** Altered metabolites of HT-29 cells after Al exposure in the negative-ion mode.

No.	Metabolites	M/Z	RT (min)	VIP value	*P* value	FC
1	Tiglic aldehyde	129.06	4.33	2.72	0.000	0.36
2	L-Lactic acid	135.03	0.71	1.20	0.006	0.36
3	Arsenobetaine	158.98	0.59	2.05	0.001	0.39
4	4-Pentenal	129.06	3.89	2.05	0.001	0.39
5	Hypothiocyanite	148.95	0.57	1.22	0.001	0.42
6	Ammonium peroxydisulfate	226.97	0.61	1.26	0.001	0.43
7	8-oxo-dGDP	442.01	1.04	1.70	0.003	0.43
8	Succinic acid semialdehyde	101.02	0.79	1.03	0.013	0.44
9	ADP	426.03	0.95	3.84	0.000	0.45
10	Uridine diphosphate glucose	565.05	0.82	1.34	0.027	0.49
11	Uridine 5′-diphosphate	403.00	0.88	2.05	0.000	0.50
12	Deoxycytidine	272.09	1.02	1.02	0.003	0.53
13	dCDP	386.02	0.75	1.28	0.005	0.54
14	Glutathione	306.08	1.02	7.14	0.002	0.55
15	Phosphoribosyl-AMP	540.05	1.03	1.63	0.000	0.55
16	D-Fructose	225.06	0.66	1.28	0.001	0.55
17	Gluconasturtiin	404.05	1.02	1.94	0.005	0.56
18	UDP-N-acetyl-alpha-D-galactosamine	606.08	0.82	4.73	0.008	0.56
19	NAD	709.11	1.04	1.04	0.019	0.57
20	UDP-D-galacturonate	579.03	0.88	4.51	0.000	0.58
21	Oxidized glutathione	611.15	1.03	4.50	0.010	0.58
22	L-Thyronine	254.08	2.92	1.06	0.008	0.58
23	Pantothenic acid	218.10	2.92	3.08	0.001	0.58
24	FAD	784.15	4.33	1.38	0.001	0.59
25	1,3,5-Trihydroxy-10-methylacridone	256.06	0.72	2.19	0.019	0.59
26	Citric acid	191.02	1.04	5.57	0.002	0.59
27	CDP	448.02	0.76	1.22	0.009	0.59
28	4-O-alpha-D-Galactopyranuronosyl-D-galacturonic acid	369.07	4.96	1.18	0.021	0.59
29	LysoPE (0:0/18:0)	480.31	11.90	1.18	0.013	0.61
30	Ibudilast	229.13	5.79	1.10	0.021	0.62
31	L-Tryptophan	203.08	4.08	1.08	0.001	0.63
32	N-Succinyl-L,L-2,6-diaminopimelate	289.10	4.95	3.07	0.027	0.63
33	5-Oxoprolinate	128.04	0.85	2.03	0.003	0.63
34	Alpha-D-Glucopyranoside	179.06	0.68	1.22	0.001	0.64
35	Succinic acid	117.02	1.32	2.02	0.028	0.64
36	Hydroxyhexamide	307.11	5.79	3.56	0.019	0.64
37	Penicilloic acid	333.09	7.33	1.95	0.021	0.65
38	2-O-p-Coumaroylhydroxycitric acid	353.05	5.94	1.96	0.012	0.66
39	L-Phenylalanine	164.07	2.18	1.48	0.010	0.66
40	Edetic acid	291.08	0.82	2.41	0.019	0.66
41	2-Aminomuconic acid	313.07	0.78	1.03	0.038	0.67
42	2-Phenylacetamide	180.07	1.17	1.33	0.006	0.67

**Notes:**

M/Z, mass/charge number of peaks in the mass spectra; RT, retention time of metabolites in chromatography; FC, fold change of metabolites after Al exposure. Differences between the control and Al-treated cells were analyzed by one-way analysis of variance, followed by the Tukey’s post hoc test. *P* < 0.05 was considered as significant. The changes in metabolite abundance are expressed as the ratio of the average content in the treatment and control groups (*n* = 6). A value < 1 indicates downregulation.

### Pathway enrichment analysis

**Figure 4:**
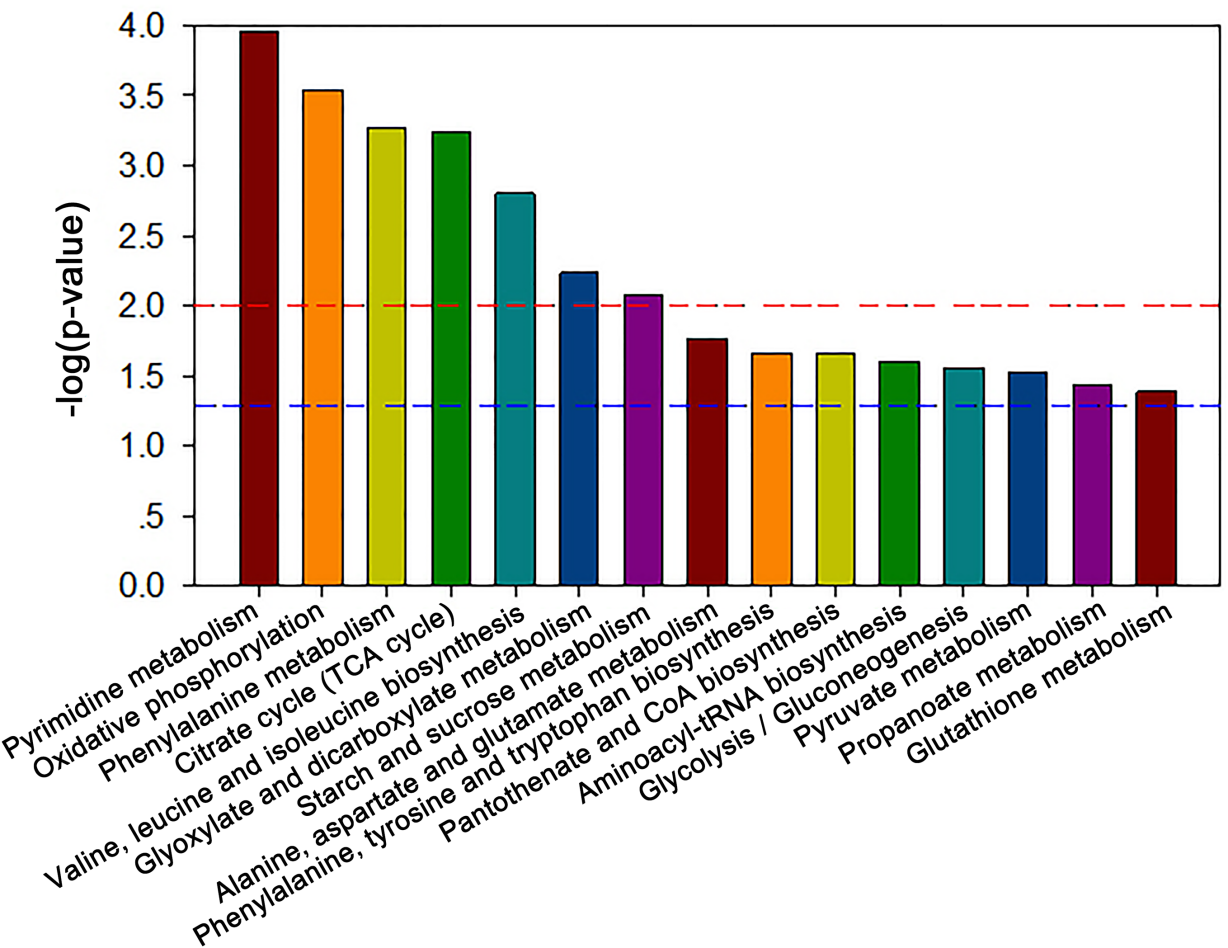
Significantly altered pathways of HT-29 cells after Al exposure in the negative-ion mode.

**Figure 5:**
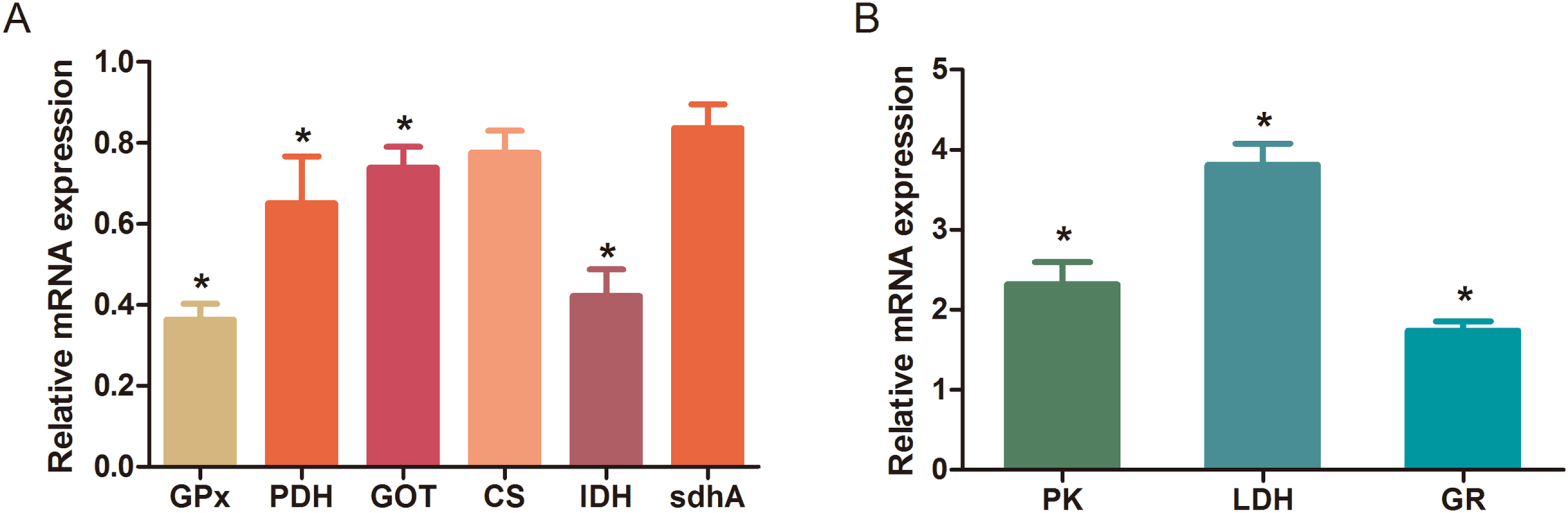
Changes in the relative mRNA expression levels of related genes after Al exposure. The values are expressed as the mean ± SD of six independent replicates with reference to the mRNA expression of the control group. (A) Downregulated genes; (B) upregulated genes. The asterisks indicate significant differences (*P* < 0.05*)*.

The MBRole-ID conversion was used to identify metabolites and enrich the pathways in KEGG. One pathway in the positive-ion mode, namely arginine and proline metabolism, and 17 pathways in the negative-ion mode, including pyrimidine metabolism, the TCA cycle, pyruvate metabolism, and GSH metabolism, were ultimately identified (all *P* < 0.05, Fig. 4). Moreover, some metabolites, such as pyruvate, citrate, succinate, leucine, tryptophan, phenylalanine, glutamate, were significantly reduced after Al exposure. The results of RT-qPCR shown in Fig. 5 (Dataset S3) presented the relative gene expression levels in the Al-treated group compared with those in the control group. Specifically, the expression of GR, PK, and LDH genes was significantly upregulated, whereas that of the other four genes (GPx, PDH, GOT, and IDH) was significantly downregulated (*P* < 0.05).

## Discussion

**Figure 6:**
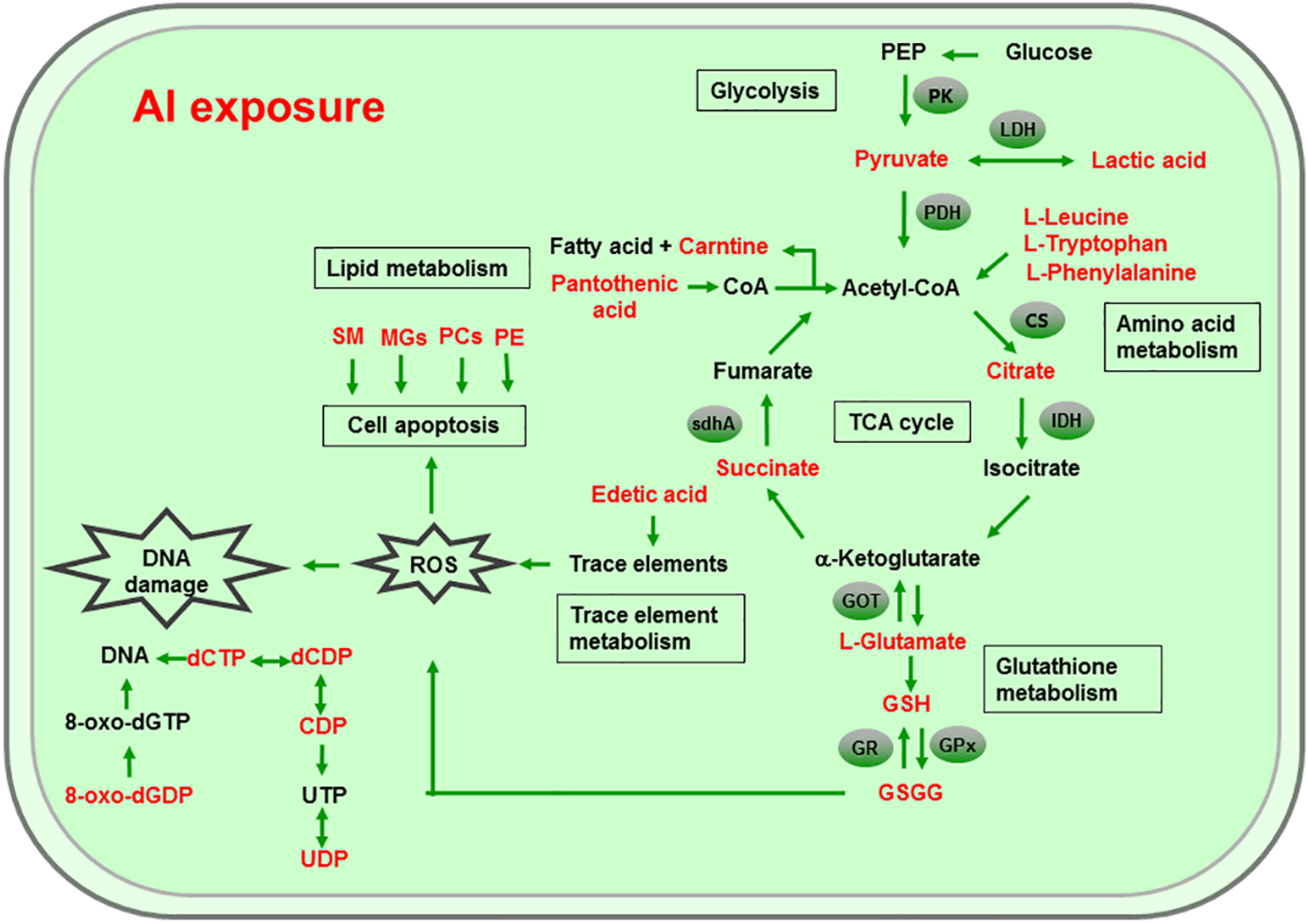
Global reactions in HT-29 cells after Al exposure.

Al ingestion caused intestinal injuries by exacerbating intestinal inflammation and damaging intestinal barrier function (Pineton de Chambrun et al., 2014). To elucidate the specific impact of Al exposure on the human intestinal tract, the HT-29 cell line was chosen as an in vitro model to investigate the global profiles of various metabolic pathways, metabolites, and genes after Al treatment. The following processes were found to be the most significantly altered: the TCA cycle; glycolysis/gluconeogenesis; GSH metabolism; pyruvate metabolism; amino acid metabolism; and biosynthesis, including those of glutamate, leucine, phenylalanine, and tryptophan; pyrimidine metabolism; and pantothenate and CoA biosynthesis (Fig. 6).

### Effects of Al cytotoxicity on energy metabolism

The TCA cycle is a common pathway in all aerobic organisms used to produce energy through the oxidation of acetyl-CoA derived from fats, carbohydrates, and proteins into ATP and CO_2_. It also provides precursors for the biosynthesis of certain amino acids and NADH. Pyruvate dehydrogenase catalyzes the decarboxylation of pyruvate, the end product of glycolysis, into acetyl-CoA. The RT-qPCR results showed that the expression of PDH in Al-treated cells was decreased to 0.65-fold of that in the untreated cells, which consequently decreased the formation of acetyl-CoA, resulting in the shortage of substrate for the TCA cycle. Consequently, the levels of intermediate compounds in the TCA cycle, such as citrate and succinate, decreased after Al exposure. Furthermore, decreased citrate and succinate levels in turn reduced NADH production, which is essential for the delivery of electrons to oxygen through the electron transport chain to produce ATP. Thus, an important mechanism of Al toxicity appears to be the perturbation of mitochondrial ATP production. These results indicate that Al exposure has a deleterious effect on the energy output and causes various cellular abnormalities. Another reason that explains the decrease in some components of the TCA cycle is the oxidation of the TCA cycle enzymes due to Al exposure. The TCA cycle enzymes are very sensitive to oxidative stress (Tretter & Adam-Vizi, 2005). Indeed, the relative mRNA expressions of IDH and GOT in Al-treated cells were found to be significantly decreased to 0.42- and 0.74-fold of those in the untreated cells, respectively (Fig. 5). Kil et al. (2006) reported that heavy metal exposure decreased IDH levels in human embryonic kidney 293 cells, impairing the conversion of citrate into isocitrate. Murakami & Yoshino (2004) found that Al inhibited IDH expression, thereby decreasing GSH regeneration in mitochondria. Benderdour et al. (2004) reported decreased IDH expression as a marker of oxidative stress in cardiac hypertrophy development.

Cell growth requires glycolysis for ATP generation to maintain the energy supply and for the accumulation of glycolytic intermediates to meet the needs of rapid cell proliferation and the need for rapid synthesis of nucleotides, lipids, and proteins (Ye et al., 2012). In the oxidative environment, cells can use glycolysis for ATP production, without the participation of mitochondria. The function of this metabolic route is crucial to maintain the energy balance of organisms in various conditions (Dang & Semenza, 1999; Kim et al., 2006). Mailloux & Appanna (2007) reported that glycolysis increases with the increase in oxidative stress in Al-treated cells. Two enzymes, PK and LDH, which are critical for ATP production in the anaerobic environment, exhibited dramatically increased activity upon Al exposure in HT-29 cells in our study. The study by Mailloux & Appanna (2007) reported similar results for Al-treated HepG2 cells. Lactic dehydrogenase is also required for the recycling of NAD^+^, which is essential for activating the subsequent step of glycolysis (Dang & Semenza, 1999).

### Effects of Al cytotoxicity on oxidative stress and amino acid metabolism

The main mechanism of Al toxicity is the induction of oxidative stress by producing excessive reactive oxygen species (ROS) (Kumar, Bal & Gill, 2009). Excess ROS levels cause imbalances and disturbances in signaling processes, such as MAPK signaling pathways, leading to growth inhibition and damage to human cells (Sharma & Dietz, 2009). Reactive oxygen species are also responsible for exacerbating DNA damage (Santos et al., 2010). Glutathione can protect cellular components against oxidative injury by directly neutralizing ROS or functioning as a cofactor against free radicals in cells (Carrola et al., 2016). In addition, being a thiol compound, GSH can bind to free metal ions with high affinity and reduce metal availability, thus indirectly reducing ROS production and alleviating oxidative stress (Sharma & Dietz, 2009). In our study, GSH level was lower in Al-treated cells than in untreated cells, suggesting that Al exposure triggered oxidative stress. Our findings also revealed decreased levels of glutamate, a GSH precursor, in the Al-treated cells, supporting the hypothesis of deregulating GSH synthesis. Glutathione peroxidase catalyzes the oxidation of GSH to GSSG, whereas GR catalyzes the reduction of GSSG to GSH. Our RT-qPCR results showed that GPx expression was reduced by 0.36-fold, whereas GR expression was increased by 1.74-fold in Al-treated cells compared with untreated cells (Fig. 5). This result indicates that the decrease in GPx expression and increase in GR expression may be a mechanism to alleviate the Al exposure-induced oxidative stress in Al-treated cells.

Upon exposure to toxins, such as toxic metals, several species downregulate or upregulate the synthesis of diverse metabolites and cause the conversion of specific amino acids. For example, glycine, alanine, serine, tryptophan, cysteine, and threonine can be converted to pyruvate through the TCA cycle, whereas aspartate and asparagine are converted to oxaloacetate (Owen, Kalhan & Hanson, 2002). Our results showed decreased intracellular levels of the ketogenic amino acids leucine, tryptophan, and phenylalanine and the glycogenic amino acid glutamate in Al-treated HT-29 cells, consistent with the results of a previous study on human epidermal keratinocytes (Carrola et al., 2016). One previous study reported that the metabolism of glutamine, the most abundant naturally occurring amino acid in the body, is upregulated during the glucose shift to provide substrate for increased lipogenesis and nucleic acid biosynthesis crucial for cell proliferation (Dakubo & Safarina, 2010). After the conversion during the glucose shift, it may be transformed into α-ketoglutarate and enter the TCA cycle. Thus, it can be speculated that decrease of amino acid level may be an indirect cause for energy and lipids metabolism alterations induced by Al exposure.

### Effects of Al cytotoxicity on lipid metabolism and cellular apoptosis

Phospholipids, such as PCs, PEs, monoglyceride, and sphingomyelin (SM), are indispensable components of cellular membranes, signaling pathways, and cellular processes such as cell–cell interactions, cell proliferation, cell differentiation, and cellular apoptosis (Hla & Dannenberg, 2012). Membrane lipids containing polyunsaturated fatty acids are highly sensitive to free radicals and are susceptible to lipid peroxidation. Our results revealed a decrease in choline and phosphocholine levels in Al-treated cells, suggesting that cell membrane disruption caused by Al exposure and the corresponding oxidative stress. In particular, the levels of PCs, LysoPCs, and PEs—crucial cellular membrane constituents—in Al-treated cells decreased to 0.28–0.64-fold of those in untreated cells (Tables 2 and 3), suggesting the degradation of membrane phospholipids and cellular apoptosis. The level of SM (d18:1/14:0) in Al-treated cells decreased to 0.43-fold of that in untreated cells, also indicating the occurrence of cellular apoptosis. A decrease in the levels of creatine, which plays a key role in energy transport across the mitochondrial membrane. Notably, a decrease in choline levels can disturb transmethylation and may also result in a decline in creatine levels. Lower carnitine level was also observed after Al treatment. A decrease in the carnitine level has been reported to cause accumulation of endotoxins and disruption of several enzyme reactions (Coulter, 1991; Russell, 2007). Carnitine deficiency is implicated in various conditions such as diabetes mellitus, Alzheimer’s disease, and heart failure (Ha et al., 2012; Li et al., 2015). After CoA biosynthesis from the precursor pantothenate, CoA is combined with a long-chain fatty acid to produce acetyl-CoA that enters the TCA cycle. Thus, disruption in the CoA biosynthesis pathway is closely related to abnormalities in energy metabolism and fatty acid biosynthesis, as well as imbalances in various cellular processes. Consequently, the availability of pantothenate plays a key role in this pathway, as indicated by a previous finding that the rate of the CoA production step is limited by the phosphorylation of pantothenate. Previous studies demonstrated that the lack of pantothenate has a severe impact on energy production and fatty acids synthesis (Rock et al., 2000).

### Other effects of Al cytotoxicity

The level of edetic acid, a common metal chelator, in Al-treated cells decreased to 0.66-fold of that in the untreated cells (Table 3). A reduction in edetic acid levels would affect the trace elements levels. Consistent with this result, Al exposure reduces the Fe, Ca, and Mg levels in mice tissues, as well as Cu, Zn, Fe, and Ca levels in humans serum (Yu et al., 2017; Metwally & Mazhar, 2007). Taken together, our results indicate that a decrease in GSH levels and disturbance in trace element levels may be the main mechanisms of Al-induced oxidative stress in HT-29 cells.

The level of 8-Oxo-dGDP, generated by the oxidation and phosphorylation of dGTP, in Al-treated cells decreased to 0.43-fold of that in the untreated cells (Table 2), suggesting that Al exposure led to nucleotide damage directly (Hayakawa et al., 1995). Moreover, Al exposure would reduce the amount of metabolites that can metabolize toxic substances. For example, 4-hydroxymidazolam level reduced to 0.65-fold after Al treatment. It is an active metabolite of midazolam, can further produce to glucuronide, metabolizing several exogenous and endogenous toxic substances and thus exerting positive and protective effects (Seo et al., 2010).

## Conclusions

In conclusion, metabolite analysis of the HT-29 cells revealed significant alterations in the TCA cycle, pyruvate metabolism, GSH metabolism, and several metabolite levels after Al exposure. Al exposure in our study led to cellular apoptosis, induced oxidative stress, altered the metabolism of energy, lipids, and amino acids. These findings explain the mechanisms of toxic effects of Al exposure in the human gastrointestinal tract.

## Supplemental Information

10.7717/peerj.7524/supp-1 Supplemental Information 1 Raw data: Cell viability of HT-29 cells after Al exposure. Viability is expressed as a cell activity percentage between the Al group and the control group.

10.7717/peerj.7524/supp-2 Supplemental Information 2 The altered metabolites of HT-29 cells after Al exposure.

10.7717/peerj.7524/supp-3 Supplemental Information 3 The changes in relative mRNA expression levels of related genes after Al exposure.

10.7717/peerj.7524/supp-4 Supplemental Information 4 Representational base peak intensity chromatograms in the positive-ion mode (A) and negative-ion mode (B).

10.7717/peerj.7524/supp-5 Supplemental Information 5 Cross-validation of the PLS-DA model in the positive-ion mode (A) and negative-ion mode (B).

## Additional Information and Declarations

### Data Availability

The following information was supplied regarding data availability: The raw data are available in the Datasets S1 and S2.
