# Supplementary material for: Metabolomic analysis reveals the mechanism of aluminum cytotoxicity in HT-29 cells
Source: PeerJ. 2019 Aug 27;7:e7524. doi: 10.7717/peerj.7524 (PMC6716502; doi:10.7717/peerj.7524)
Supplement: Supplemental Information 1 — Viability is expressed as a cell activity percentage between the Al group and the control group. [file peerj-07-7524-s001.docx]

**Dataset S1 Cell viability of HT-29 cells after Al exposure**

| **Cell viability (%)** | | | | | | | | | |
| --- | --- | --- | --- | --- | --- | --- | --- | --- | --- |
| CK | 1mM | 2mM | 3mM | 4mM | 5mM | 6mM | 7mM | 8mM | 10mM |
| 100 | 91.1193 | 82.48016 | 78.80386 | 46.78092 | 49.13349 | 31.27475 | 20.12746 | 8.591473 | 2.039607 |
| 100 | 87.94684 | 83.62328 | 78.56052 | 51.08928 | 40.65709 | 35.31313 | 23.47199 | 13.27443 | 2.251162 |
| 100 | 101.0955 | 87.02694 | 75.56126 | 54.6618 | 46.65985 | 28.42404 | 19.02012 | 10.3458 | 3.781522 |
