# Supplementary material for: Metabolomic analysis reveals the mechanism of aluminum cytotoxicity in HT-29 cells
Source: PeerJ. 2019 Aug 27;7:e7524. doi: 10.7717/peerj.7524 (PMC6716502; doi:10.7717/peerj.7524)
Supplement: Supplemental Information 3 [file peerj-07-7524-s003.docx]

**DatasetS3 The changes in relative mRNA expression levels of related genes after Al exposure (Fold of control).**

| GPx | IDH | PDH | GOT | CS | sdhA | GR | PK | LDH |
| --- | --- | --- | --- | --- | --- | --- | --- | --- |
| 0.40218 | 0.41 | 0.53364 | 0.73598 | 0.7233 | 0.83879 | 1.87142 | 2.36486 | 3.75099 |
| 0.36473 | 0.36253 | 0.65243 | 0.79246 | 0.77476 | 0.77782 | 1.64806 | 2.02304 | 4.10075 |
| 0.32401 | 0.4934 | 0.76659 | 0.68899 | 0.83133 | 0.89484 | 1.6991 | 2.57244 | 3.58 |
