# Supplementary figures and images for: Metabolomic analysis reveals the mechanism of aluminum cytotoxicity in HT-29 cells
Source: PeerJ. 2019 Aug 27;7:e7524. doi: 10.7717/peerj.7524 (PMC6716502; doi:10.7717/peerj.7524)

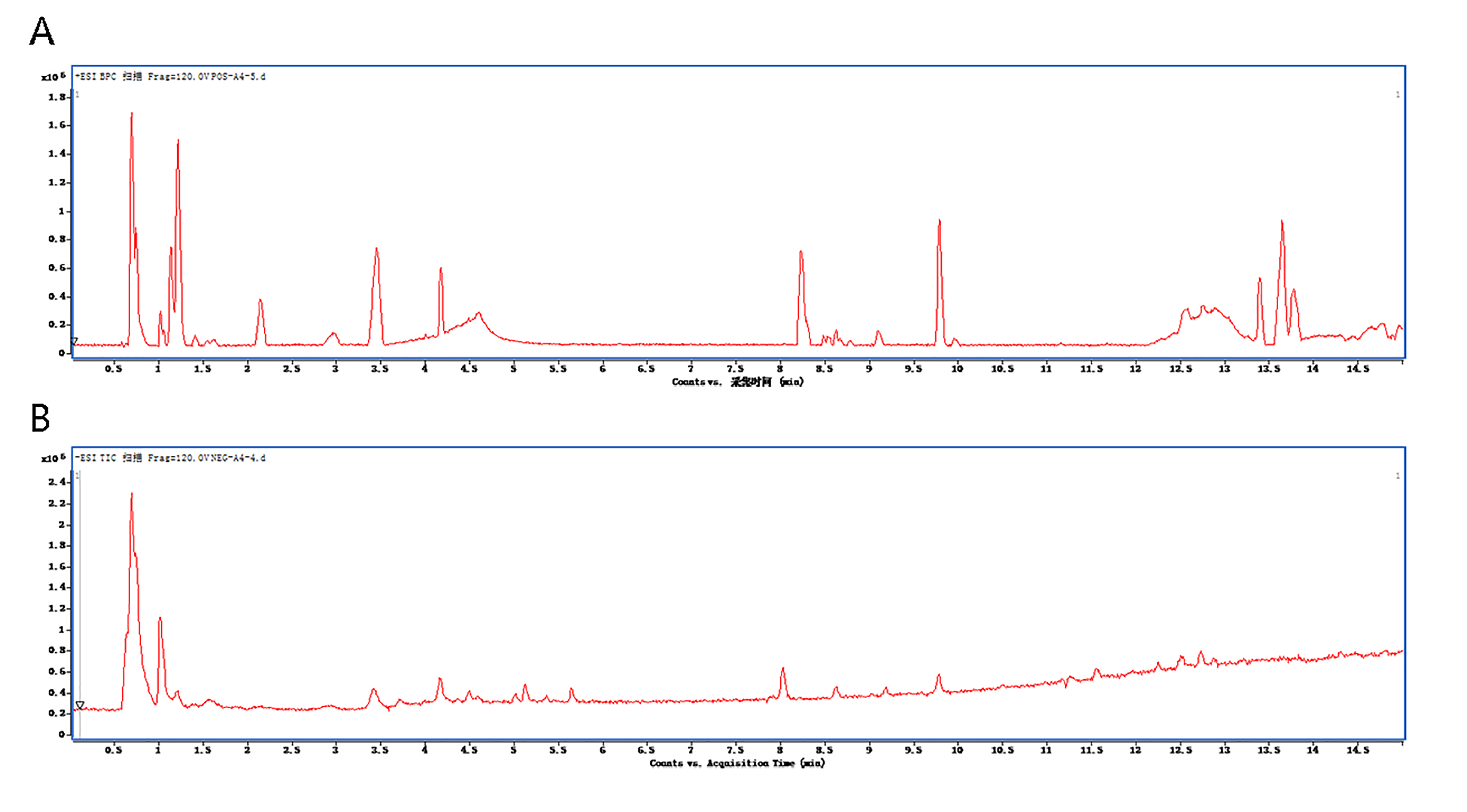

Supplement: Supplemental Information 4 [file peerj-07-7524-s004.png]

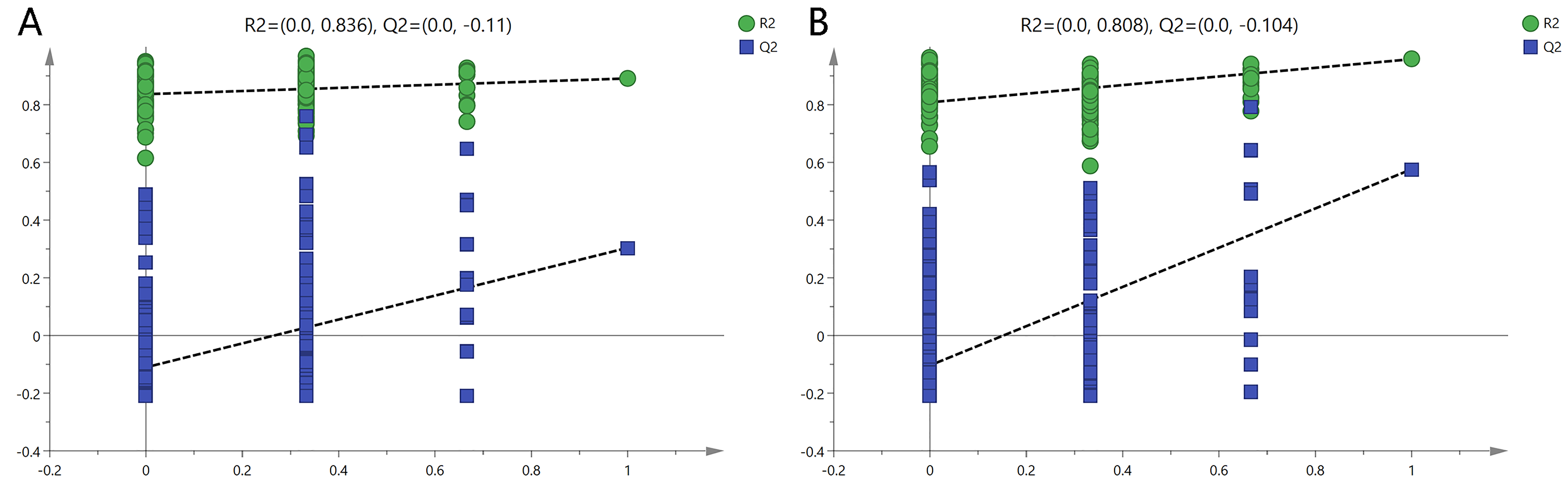

Supplement: Supplemental Information 5 [file peerj-07-7524-s005.png]
